# Trends and Patterns in Mandible Bone Cancer Research: An Updated Comprehensive Bibliometric Review: Prevalence of Supernumerary Teeth in Patients with Cleft lip and Palate

**DOI:** 10.31661/gmj.v13iSP1.3541

**Published:** 2024-12-31

**Authors:** Hamidreza Arabion, Arya Khosronejad, Alireza Afshar, Aida Iraji, Mohamad Mokhtarzadegan, Ehsan Aliabadi, Reyhaneh Ebrahimi, Nadiar M. Mussin, Madina A. Kurmanalina, Nader Tanideh, Amin Tamaddon

**Affiliations:** ^1^ Department of Oral and Maxillofacial Surgery, School of Dentistry, Shiraz University of Medical Science, Shiraz, Iran; ^2^ Student Research Committee, Bushehr University of Medical Sciences, Bushehr, Iran; ^3^ Stem Cells Technology Research Center, Shiraz University of Medical Sciences, Shiraz, Iran; ^4^ Central Research laboratory, Shiraz University of Medical Sciences, Shiraz, Iran; ^5^ School of Metallurgy and Materials Engineering, College of Engineering, University of Tehran, Tehran, Iran; ^6^ Department of Periodontics, School of Dentistry, Shiraz University of Medical Sciences, Shiraz, Iran; ^7^ Department of Surgery No. 2, West Kazakhstan Medical University, Aktobe, Kazakhstan; ^8^ Department of Therapeutic and Prosthetic Dentistry, West Kazakhstan Marat Ospanov Medical University, Aktobe, Kazakhstan; ^9^ Department of Pharmacology, School of Medicine, Shiraz University of Medical Sciences, Shiraz, Iran; ^10^ Department of Natural Sciences, West Kazakhstan Marat Ospanov Medical University, Aktobe, Kazakhstan

**Keywords:** Neoplasm, Tumor, Mandible, Mandibular Bone, Surgery

## Abstract

**Background:**

Mandible bone cancer, while relatively rare, poses significant challenges due to its aggressive nature and the complex treatment required. This bibliometric review aims to analyze the trends and patterns in mandible bone cancer research from 1933 to 2024, providing insights into the evolution of scholarly contributions and the impact of various studies in this field.

**Materials and Methods:**

Data were collected from the Web of Science Core Collection and Scopus databases, focusing on articles published between 1933 and 2024. A total of 8,093 articles were analyzed, with performance metrics evaluated using RStudio and the bibliometrix R-package. Key metrics included publication growth, citation analysis, and keyword frequency assessment.

**Results:**

The analysis revealed a steady annual growth rate of 6.05% in publications, with significant contributions from institutions such as the University of California and Tokyo Medical and Dental University. The average citation per manuscript was 18.41, indicating robust engagement with the literature. Key themes identified included mandible, mandibular reconstruction, and osteoradionecrosis, reflecting ongoing research interests and collaborative networks among authors.

**Conclusion:**

This review highlights the increasing scholarly attention on mandible bone cancer, emphasizing the need for continued research to address existing gaps in diagnosis and treatment. Collaborative efforts among institutions and researchers will be crucial in advancing the understanding and management of this complex disease, ultimately improving patient outcomes.

## Introduction

The mandible, or lower jawbone, is a fundamental component of the human skeletal system, playing a crucial role in mastication, speech, and facial aesthetics [1]. As the largest and strongest bone of the face, the mandible forms the lower jaw and holds the lower teeth in place [2]. Structurally, it comprises a horizontal body and a vertical ramus, which converge at the mandible’s angle [3]. The body of the mandible supports the lower teeth through the alveolar process, while the ramus connects to the skull at the temporomandibular joint (TMJ), facilitating jaw movement [4]. Beyond its structural and functional roles, the mandible is integral to facial symmetry and expression, significantly impacting an individual’s appearance [5]. Designed to endure the forces generated during chewing, the mandible’s robust structure also houses the inferior alveolar nerve, which provides sensation to the lower lip and chin, underscoring its role in sensory and motor functions [6].

Mandibular defects can arise from congenital conditions, trauma, infection, and neoplastic diseases [7]. These defects often lead to substantial functional and aesthetic impairments, severely affecting a person’s quality of life [8]. Congenital defects like mandibular hypoplasia present from birth, whereas traumatic injuries are more common in younger individuals due to accidents or violence [9]. Additionally, infections such as osteomyelitis can severely damage the mandibular bone, necessitating extensive medical intervention [10].

Neoplastic diseases, particularly mandibular cancers, represent some of the most severe impacts on the mandible. Although relatively rare, mandibular cancer poses a significant burden due to its aggressive nature and complex treatment [11][12]. Cancers affecting the mandible include osteosarcoma, chondrosarcoma, and metastatic cancers from primary sites like the breast, lung, or prostate [13]. Osteosarcoma of the mandible, known for its aggressive behavior and poor prognosis, underscores the importance of early detection and treatment, despite symptoms often resembling less severe conditions [14]. The burden of mandibular cancer extends beyond physical symptoms, affecting patients psychologically and socially due to the visible nature of the disease and the deformities from cancer and its treatment. Recent statistics indicate that the five-year survival rate for mandibular osteosarcoma is approximately 60%, with an annual incidence of adolescent about 6.7 per million [15]. Surgical resection, often necessary to remove tumors, can lead to significant disfigurement and functional loss. Reconstructive surgery aims to mitigate these effects but involves a complex, carefully planned process [16][17][18]. Additionally, the financial burden on patients and healthcare systems is substantial, covering direct treatment costs and long-term care and rehabilitation [19].

Treating mandibular cancer typically involves a multidisciplinary approach, including surgery, radiation therapy, and chemotherapy [20][21][22]. Surgical treatment aims to remove the tumor and affected tissues, followed by reconstructive surgery to restore function and aesthetics [23]. Advances in surgical techniques, like vascularized bone grafts, have significantly improved outcomes for mandibular reconstruction patients [24]. Radiation therapy is often used alongside surgery to eliminate residual cancer cells and reduce recurrence risk [25]. Chemotherapy is considered for metastatic or inoperable cancer cases [6]. Reconstructive techniques have advanced, with microvascular free flaps now the gold standard for mandibular reconstruction. These flaps, which include tissues from other body parts with their blood supply, provide a reliable method for reconstructing large defects [10]. The fibula free flap is particularly favored for its length and bone quality, allowing for dental implant placement [26]. Additionally, cell therapy, including the stem cells, is being explored as a novel approach to target and eradicate cancer cells [27][28]. Despite these advancements, reconstructive surgery remains challenging and requires expert coordination among surgical teams [9].

Research into mandible bone cancer is vital. Bibliometric studies of trends and patterns in this field offer valuable insights into research progress and direction [29]. Such analysis helps quantify the impact of various studies, identify leading contributors, and uncover collaborative networks across institutions and countries. This, in turn, aids in identifying research gaps and prioritizing future research efforts [30][31]. Advances in treatment and reconstruction techniques of mandible defects, exclusively mandibular bone cancer, have improved outcomes, yet ongoing research is crucial for continued progress [14]. Bibliometric studies play a key role by providing a comprehensive overview of research activities, guiding future studies, and enhancing our understanding and treatment of mandible bone cancer [30][31].

## Materials and Methods

**Table:** Table1. Queries of search strategy in Scopus and Web of Science (WOS)

**#1**	"Neoplasms" OR "Tumor" OR "Neoplasm" OR "Tumors" OR "Neoplasia" OR "Neoplasias" OR "Cancer" OR "Cancers" OR "Malignant Neoplasm" OR "Malignancy" OR "Malignancies" OR "Malignant Neoplasms" OR "Neoplasm, Malignant" OR "Neoplasms, Malignant" OR "Benign Neoplasms" OR "Benign Neoplasm" OR "Neoplasms, Benign" OR "Neoplasm, Benign" OR "Benign Neoplasm"
**#2**	"Mandible" OR "Mandibles" OR "Mylohyoid Ridge" OR "Mylohyoid Ridges" OR "Ridge, Mylohyoid" OR "Ridges, Mylohyoid" OR "Mylohyoid Groove" OR "Groove, Mylohyoid" OR "Grooves, Mylohyoid" OR "Mylohyoid Grooves"
**#3**	"Bone and Bones" OR "Bone and Bone" OR "Bones and Bone" OR "Bones and Bone Tissue" OR "Bones" OR "Bone" OR "Bone Tissue" OR "Bone Tissues" OR "Tissue, Bone" OR "Tissues, Bone" OR "Bony Apophyses" OR "Apophyses, Bony" OR "Bony Apophysis" OR "Apophysis, Bony" OR "Condyle" OR "Condyles"
**#4**	#1 AND #2 AND #3

Date of search: 26/7/2024

**Table:** Table2. Codes were used to merge Scopus and Web of Science exported data in RStudio

library(bibliometrix) library(openxlsx) ## importing web of science dataset web_data<-convert2df("abs.txt") ## importing scopus dataset scopus_data<-convert2df("abs.bib",dbsource="scopus",format="bibtex") ##combined both datasets combined<-mergeDbSources(web_data,scopus_data,remove.duplicated=T) ##exporting file write.xlsx(combined,"combinedabs.xlsx")

### Data Collection

**Figure-1:**
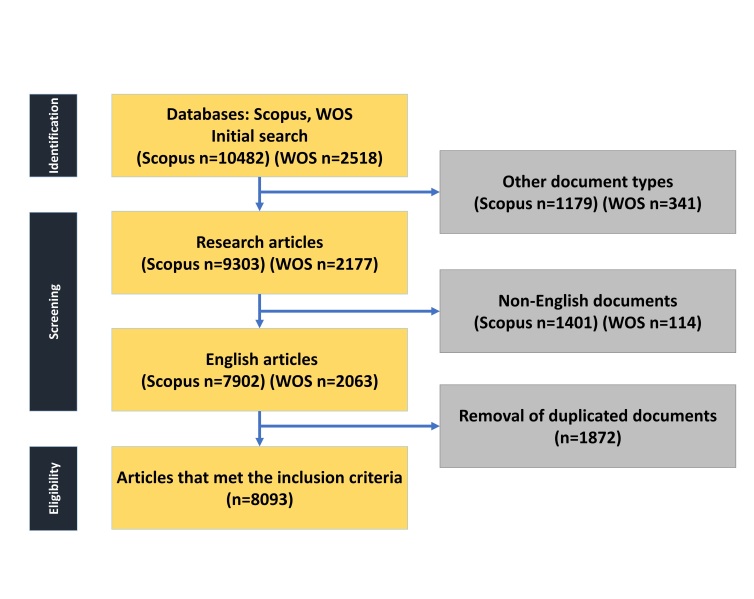
Figure 1. The article selection process from the Scopus and Web of Science (WOS) databases was illustrated through a flowchart designed in accordance with PRISMA guidelines.

In July 2024, data were sourced from the Web of Science Core Collection (WOS-CC) and Scopus databases to perform an extensive analysis of research on the impact of TQ on neoplasms. The search strategy aimed to be comprehensive, encompassing various facets of the topic (Table-1). Specific inclusion criteria were implemented to ensure data accuracy: (1) articles published between 1997 and 2024, (2) articles written in English, and (3) exclusion of review articles, proceeding papers, book chapters, and editorial material. Data extracted from both databases were merged (Table-2), verified, and duplicate entries were removed. A flowchart illustrating the data extraction process is provided in Figure-1.

### Performance Analysis

Performance analysis and science mapping were conducted using RStudio v.2024.04.0 and the bibliometrix R-package (4.2.0). Biblioshiny, an open-source package, was utilized for data analysis, which operates with a single database at a time [32]. WOS and Scopus were chosen due to their comprehensive and detailed citation information, which is essential for thorough bibliometric analysis and assessing research impact. Trends in local publications and average total citations per article were evaluated annually.

### Identification of Leading Institutions, Sources, Authors, and Collaborating Countries

The top 10 most productive institutions and authors were identified based on their proportion of authored papers. Collaboration patterns between institutions and authors were graphically represented. At the country level, the proportion of articles from each country was used to identify the most productive countries, and the proportion of multi-country collaborations was calculated for the top 10 countries. The collaboration network among countries was mapped based on the number of publications contributed by each country.

### Keywords Frequencies Analysis

A temporal analysis was conducted to track the periodic occurrence of specific keywords over the years. A TreeMap was created to illustrate the distribution and prominence of the top 10 recurring keywords. An in-depth thematic analysis was performed to highlight dominant trends and themes within the selected articles.

### Funding

The research presented in this article was supported by funding from Shiraz University of Medical Science (Grant number IR.SUMS.AEC.1401.128). The funders had no role in the study design, data collection and analysis, decision to publish, or manuscript preparation.

### Institutional Review Board Statement

The in vitro and in vivo studies were approved by a specialized ethics committee working with laboratory animals at Shiraz University of Medical Sciences (ethical code: IR.SUMS.AEC.1401.128). All study procedures were performed in accordance with the Declaration of Helsinki and the experimental rats received human care.

## Results

**Figure-2:**
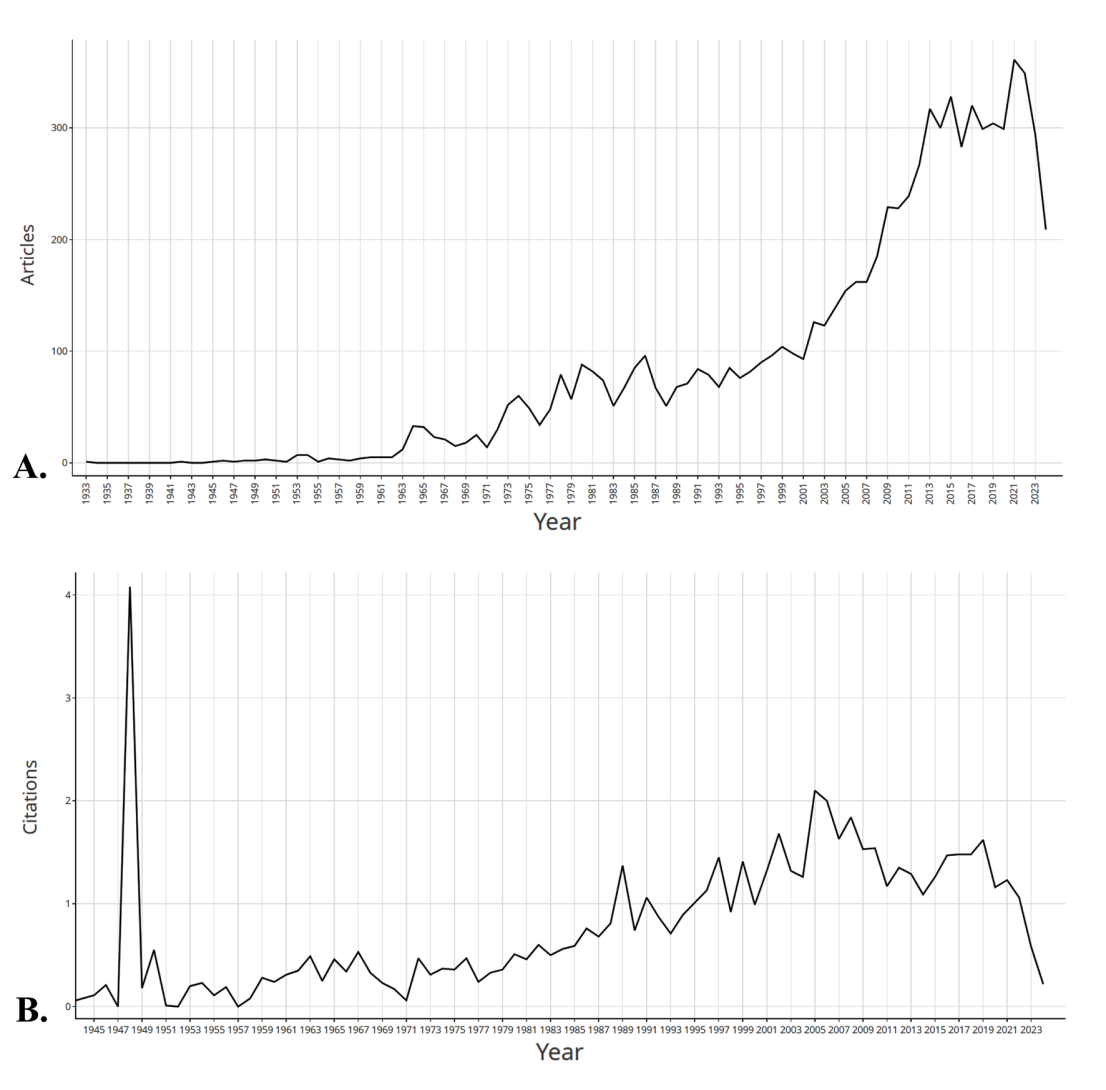
Figure 2. The global annual trends in (A) the number of publications and (B) citations related to mandible bone cancer research from 1933 to 2024 were analyzed.

**Figure-3:**
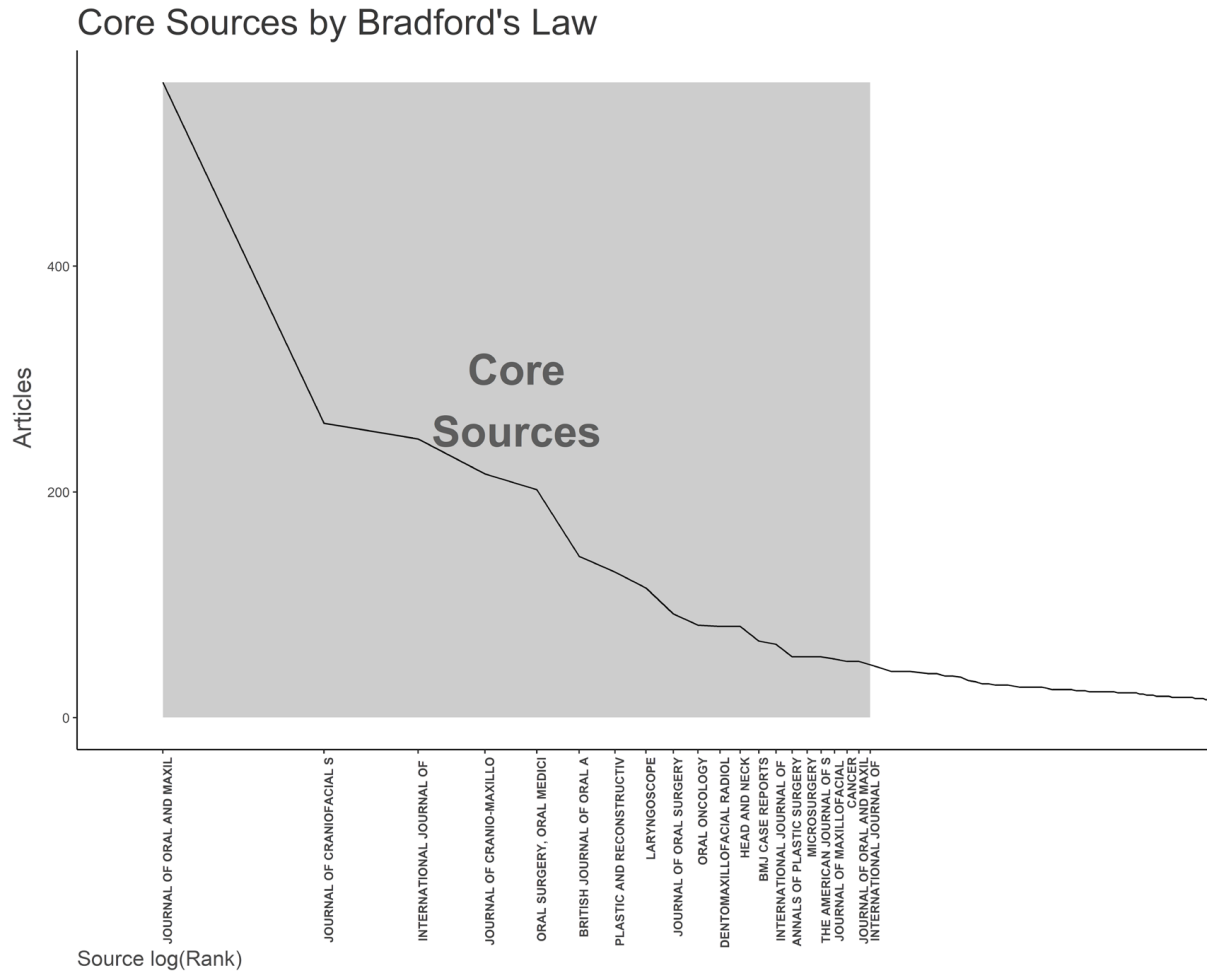
Figure 3. The application of Bradford’s Law was demonstrated, identifying twelve key journals that are central to the field of mandible bone cancer research from 1933 to 2024.

**Figure-4:**
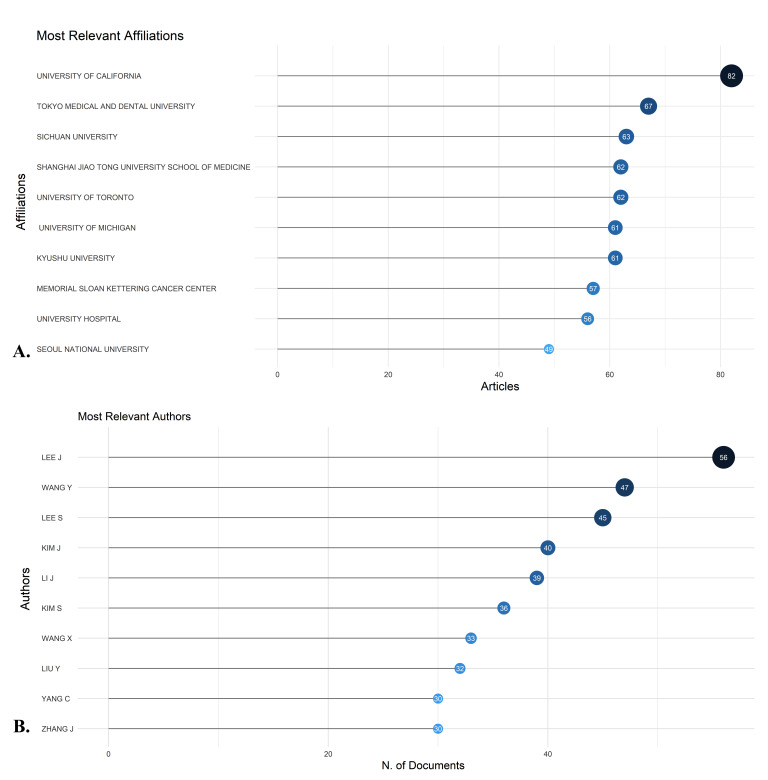
Figure 4. The analysis presented the most productive authors, institutions, and countries, along with their collaboration networks (A). Additionally, the ten authors who contributed most significantly to this field and their publication output over time regarding mandible bone cancer research from 1933 to 2024 were highlighted (B).

**Table:** Table3. The ten most referenced documents on the mandible bone cancer research (1933-2024).

**Rank**	**Study ID [References]**	**Title of the Document**	**Journal Name**	**Total** **Citations**	**DOI/PMID**
1	Hidalgo Da, 1989 (68)	Fibula free flap: a new method of mandible reconstruction	Plastic and Reconstructive Surgery	1365	10.1097/00006534-198907000-00014
2	Marx Re, 2005 (69)	Bisphosphonate-induced exposed bone (osteonecrosis/osteopetrosis) of the jaws: risk factors, recognition, prevention, and treatment	Journal of Oral and Maxillofacial Surgery	1127	10.1016/j.joms.2005.07.010
3	Coleman Sr, 2006 (70)	Structural fat grafting: more than a permanent filler	Plastic and Reconstructive Surgery	1050	10.1097/01.prs.0000234610.81672.e7
4	Ackerman Lv, 1948 (71)	Verrucous carcinoma of the oral cavity	Surgery	616	10.5555/uri:pii:S0039606048901056
5	Swartz Wm, 1986 (72)	The osteocutaneous scapular flap for mandibular and maxillary reconstruction	Plastic and Reconstructive Surgery	598	10.1097/00006534-198604000-00003
6	Wei Fc, 1994 (73)	Fibula osteoseptocutaneous flap for reconstruction of composite mandibular defects	Plastic and Reconstructive Surgery	394	10.1097/00006534-199402000-00009
7	Hirshberg A, 2008 (74)	Metastatic tumours to the oral cavity - Pathogenesis and analysis of 673 cases	Oral Oncology	386	10.1016/j.oraloncology.2007.09.012
8	Murphey Md, 2001 (75)	Imaging of giant cell tumor and giant cell reparative granuloma of bone: radiologic-pathologic correlation	RadioGraphics	377	10.1148/radiographics.21.5.g01se251283
9	Jewer Dd, 1989 (76)	Orofacial and mandibular reconstruction with the iliac crest free flap: a review of 60 cases and a new method of classification	Plastic and Reconstructive Surgery	365	10.1097/00006534-198909000-00001
10	Migliorati Ca, 2005 (77)	Bisphosphonate-associated osteonecrosis of mandibular and maxillary bone	Cancer	364	10.1002/cncr.21130

**Table:** Table4. Lists the ten most cited journals on the topic of the mandible bone cancer research (1933-2024).

**Country**	**Number of Articles**
Journal of Oral and Maxillofacial Surgery	1617 **2**
Plastic and Reconstructive Surgery	9675
International Journal of Oral and Maxillofacial Surgery	5823
Journal of Cranio-Maxillofacial Surgery	5648
Oral Surgery, Oral Medicine, Oral Pathology	4797
Cancer	3919
Laryngoscope	3887
Oral Oncology	3257
British Journal of Oral and Maxillofacial Surgery	2853
Head and Neck	2580

### Comprehensive Overview of the Manuscripts

This study aims to provide a thorough analysis of global scholarly contributions on the topic of mandible bone cancer research, covering articles published from 1933 to 2024. A total of 8093 relevant studies were carefully examined, originating from 1601 different sources. Contributions from 23981 researchers were included, resulting in an average of 18.41 citations per manuscript over the past decades. The most frequently cited documents on this topic are highlighted in Table-3, illustrating key findings related to the subject of mandible bone cancer. The Annual Growth Rate for this field was calculated to be 6.05%, indicating a steady increase in publications over the study period. The significant research output is further emphasized by the inclusion of 31413 references and 8182 unique author keywords. A total of 2.39% proportion of authors participated in collaborative studies.

### Evolution of Publication and Citation Metrics

An analysis of the dataset reveals notable fluctuations in the average cumulative citations per manuscript across the studied timeline. A significant increase was observed in 1948, where the average cumulative citations per manuscript reached a peak of 314.5. Conversely, 1933, 1947 and 1952 exhibited the lowest average cumulative citations, with a value of zero. Thise value for recent two decades were highest for 2005 with 42.03 of average cumulative citations per manuscript. The volume of publications (N) also varied annually, with the highest number of articles being published in 2022 (N = 361), and the lowest recorded in the years 1933, 1942, 1945, 1947, 1952 and 1955 (N = 1). These trends in publication over time are illustrated in Figure-2.

Applying Bradford’s Law, which describes the distribution of scholarly articles across different journals, we identified 21 core journals that are preferred by researchers in this field (Figure-3). These core journals represent a significant portion of the total articles published on the mandible bone cancer research. Notably, "Journal of oral and maxillofacial surgery" emerged as the most prolific journal, contributing 563 articles, approximately 6.95% of the total articles during the study period. Furthermore, an analysis of local citations within these core journals revealed that the mentioned journal, "Journal of oral and maxillofacial surgery", garnered the highest number of local citations, totaling 16172 (Table-4).

### Most Productive Authors, Institutions, Countries, and Their Collaboration Network

**Figure-5:**
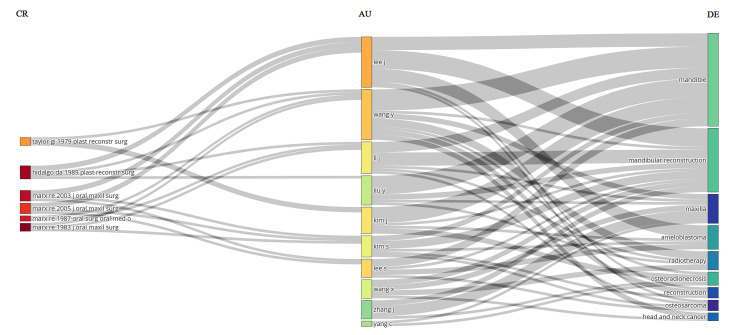
Figure 5. A Three-Fields Plot was utilized to depict the relationships among cited references (CR), authors (AU), and author keywords (DE) associated with mandible bone cancer research from 1933 to 2024

The analysis identified the University of California, Tokyo Medical and Dental University, Sichuan University, Shanghai Jiao Tong University School of Medicine, University of Toronto, University of Michigan, Kyushu University, Memorial Sloan Kettering Cancer Center, University Hospital, and Seoul National University as the most productive institutions. These institutions contributed 82, 67 , 63, 62, 62, 61, 61, 57, 56, and 49 articles, respectively (Figure-4A). Among individual authors, Lee J. was the most prolific with 56 articles (0.14%), followed by Wang Y. with 47 articles (Figure-4B). The Three-Fields Plot (Figure-5) highlights the complex network of citations, authors, and keywords, providing a comprehensive overview of the scholarly landscape on the topic of mandible bone cancer research from 1933 to 2024.

**Figure-6:**
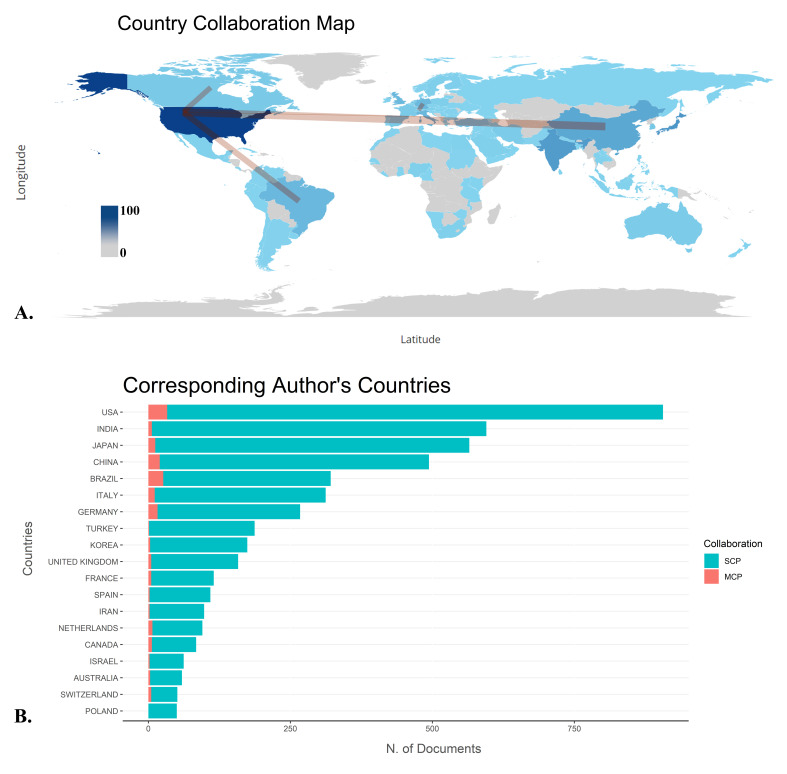
Figure 6. The figure depicted a collaboration map among countries and the nations of corresponding authors in the context of mandible bone cancer research from 1933 to 2024. This word map serves as a global overview of collaboration in the field (A), with color saturation indicating the volume of articles produced by each country. The thickness of the connecting arrows represented the strength of collaboration between nations. The most significant countries of origin for corresponding authors were also highlighted (B). Publications from single countries (SCP) and those involving multiple countries (MCP) were represented in green and orange, respectively.

USA led the global scientific output with 906 publications, followed by the India with 595 publications, Japan with 565, China with 494, and Brazil with 321 (Table-5). USA showed a strong preference for single-country publications, accounting for 96.3% of its total output, while the India had 98.9% of its publications as single-country studies. However, USA, Brazil and China had the highest number of collaborative publications with other countries, with 33, 26 and 20 publications, respectively. The primary strength of international collaboration was observed between China, the USA (Figure-6).

### Co-occurrence, Focal Points, and Evolving Keywords

**Figure-7:**
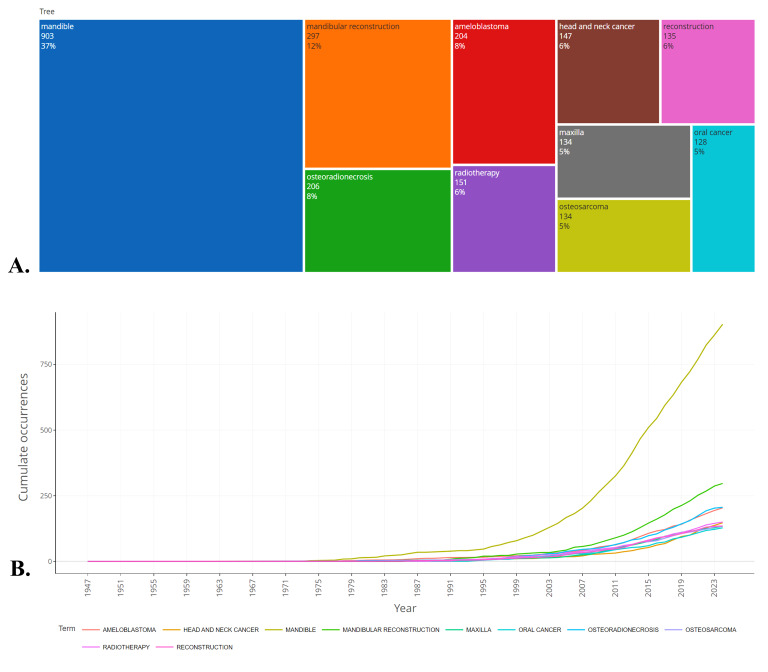
Figure 7. The study included a TreeMap (A) and a scatter plot (B) that illustrated the top ten author keywords related to mandible bone cancer research from 1933 to 2024.

Utilizing Biblioshiny, the analysis of frequently occurring author keywords revealed a focus on terms such as mandible, mandibular reconstruction, osteoradionecrosis, and ameloblastoma. The keyword "mandible" showed an increasing trend, with 903 occurrences in 2024; following by mandibular reconstruction, osteoradionecrosis, and ameloblastoma with 297, 206 and 204 occurrences in 2024. The keyword "oral cancer" maintained a relatively constant frequency over the years, recording 128 instances in 2024 (Figure-7).

**Figure-8:**
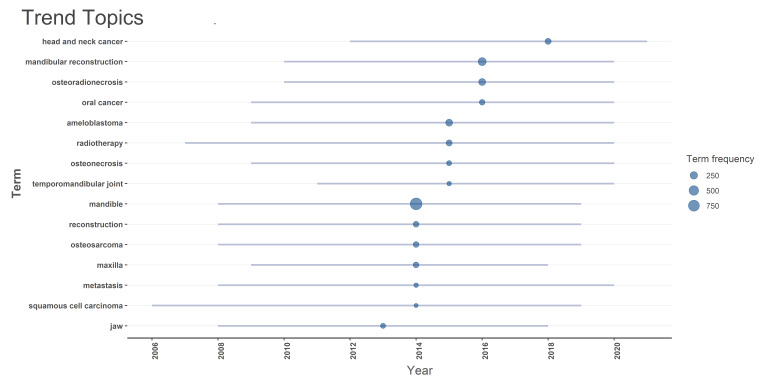
Figure 8. A timeline was presented to depict trending topics in mandible bone cancer research from 1933 to 2024. Each bubble indicated the peak frequency of keyword usage, while the accompanying line illustrated the years in which these keywords were most frequently employed.

The timeline analysis of key terms indicates that " mandible " and " mandibular reconstruction" reached their peak citations in 2014 and 2016, respectively, with 903 and 297 of the citations. Other significant terms like "osteoradionecrosis" (206) and "ameloblastoma" (204) also had high frequencies in 2016 and 2015 respectively, reflecting ongoing interest in understanding the mandible bone cancer, its structure and reconstruction (Figure-8).

## Discussion

**Table:** Table5. Ranks the leading countries publishing on the mandible bone cancer research (1933-2024).

**Country**	**Number of Articles**	**Single countries publication (SCP) **	**Multiple countries publication (MCP) **
USA	906	873	33
INDIA	595	589	6
JAPAN	565	553	12
CHINA	494	474	20
BRAZIL	321	295	26
ITALY	312	301	11
GERMANY	267	251	16
TURKEY	187	186	1
KOREA	174	171	3
UNITED KINGDOM	158	153	5

### Bibliometrics analysis findings

Bibliometric research is grounded in the meticulous analysis of publication metadata, encompassing a wide range of elements such as author affiliations, publication types, geographical origins, funding information, and citation details. In the current era of knowledge advancement, there has been a notable acceleration in the fields of bibliometric analysis and scientific mapping. This surge can be largely attributed to the growing interest and enthusiasm within the scientific community to harness the valuable insights and knowledge gained from diverse bibliometric studies [33].

This bibliometric review of mandible bone cancer research from 1933 to 2024 revealed significant trends and patterns in scholarly output, highlighting the growing interest in this area of study. A total of 8,093 articles were identified, reflecting contributions from 23,981 authors across 1,601 sources. The average citation per manuscript was 18.41, indicating a robust engagement with the literature. The annual growth rate of publications was calculated at 6.05%, suggesting a steady increase in research activity over the decades. These findings are consistent with previous studies that have documented an upward trend in research output related to head and neck cancers [29]. For instance, a study by Lee et al. (2023) noted a similar increase in publications concerning oral cancers, with a focus on the need for improved diagnostic and therapeutic strategies [34]. The prominent institutions contributing to this research included the University of California and Tokyo Medical and Dental University, which aligns with findings of previous studies, who reported these institutions as leaders in oral and maxillofacial surgery research [34][35]. Keyword analysis revealed a concentration on terms such as "mandible," "mandibular reconstruction," and "osteoradionecrosis," with the term "mandible" appearing most frequently. This trend mirrors the findings of previous bibliometric analysis [34][35], which highlighted similar keyword frequencies in the context of oral cancer research, suggesting that these areas are of significant interest to researchers.

### Bibliometrics analysis limitations

While bibliometric analyses provide a quantitative overview of research trends, they have limitations in advancing the field’s conceptual understanding [36]. These methods often prioritize publication metrics like citation counts and volume over assessing the depth and quality of scholarly contributions, such as theoretical advancements and methodological innovations [37]. As a result, bibliometric studies may overlook crucial aspects of research content, including the robustness of methods, the quality of evidence, and theoretical contributions, which are essential for a thorough evaluation of scientific progress [38]. The emphasis on quantitative metrics can also introduce biases, as highly cited research does not always equate to the most innovative or impactful work [39]. Furthermore, bibliometric methods frequently fail to capture the value of interdisciplinary research accurately, thus neglecting significant contributions that do not align with traditional citation databases [40]. Therefore, while these methods are useful for identifying general patterns, they should be complemented with qualitative assessments to gain a comprehensive understanding of research impact and quality [41].

To address these limitations, the current study conducted an in-depth literature review that significantly enhances the manuscript’s contribution by providing a more nuanced and comprehensive analysis of the research topic. The qualitative approach allows for a deeper exploration of the conceptual underpinnings, methodological rigor, and theoretical implications of the existing literature, which is crucial for advancing the field’s understanding. By combining quantitative and qualitative assessments, this study aims to present a more balanced and insightful perspective on the current state of research and identify areas for future exploration.

### Literature Review on Mandibular Defects and Mandibular Cancers

Mandibular defects can arise from various etiologies, including congenital anomalies, traumatic injuries, infections, and neoplastic diseases. Congenital defects, such as mandibular hypoplasia, are present at birth and can significantly impact function and aesthetics [12][42]. Traumatic injuries, often seen in younger populations due to accidents or violence, can lead to substantial bone loss and functional impairment [43]. Infections like osteomyelitis can also compromise the integrity of the mandible, necessitating complex medical interventions [44][45]. Neoplastic diseases, particularly cancers of the mandible, represent a critical area of concern due to their aggressive nature and treatment challenges. Mandibular cancers, including osteosarcoma and chondrosarcoma, are relatively rare but can have severe implications for patient health and quality of life [46][47]. The burden of these cancers extends beyond physical symptoms, as they can lead to significant psychological and social challenges due to visible deformities and functional limitations [48][49].

### Diagnostic Approaches for Mandibular Cancers

Accurate diagnosis is paramount for effective management of mandibular cancers. Imaging techniques, including computed tomography (CT) and magnetic resonance imaging (MRI), are essential for assessing tumor size, location, and extent of involvement. CT scans provide detailed visualization of bony structures, while MRI is particularly useful for evaluating soft tissue infiltration [50][51]. Positron emission tomography (PET) scans can also play a role in detecting metastasis and monitoring treatment response [50]. Biopsy remains the gold standard for confirming a diagnosis of mandibular cancer. Techniques such as fine-needle aspiration (FNA) and core needle biopsy allow for the collection of tissue samples for histopathological examination [6][52][53]. Recent advancements in molecular diagnostics and immunohistochemistry have enhanced the ability to classify tumors and tailor treatment strategies based on specific tumor characteristics [52][54].

### Treatment Strategies and Challenges

The management of mandibular cancers typically involves a multidisciplinary approach, incorporating surgery, radiation therapy, and chemotherapy. Surgical resection is often necessary to remove the tumor and any affected tissues, followed by reconstructive surgery to restore function and aesthetics [55][56]. Advances in surgical techniques, such as the use of vascularized bone grafts and microvascular free flaps, have improved outcomes for patients undergoing reconstruction [24][26]. Radiation therapy is frequently employed in conjunction with surgical intervention to eliminate residual cancer cells and reduce the risk of recurrence [22]. Chemotherapy may be indicated for patients with metastatic disease or those who are not candidates for surgery [57]. Emerging targeted therapies based on the molecular profile of tumors are also being explored as potential treatment options [58]. Stem cell therapy, particularly the use of mesenchymal stem cells for bone regeneration, is another promising area being investigated to enhance reconstruction outcomes and mitigate complications [59][60]. Despite these advancements, the treatment of mandibular cancers presents several challenges. The complex anatomy of the mandible and its proximity to vital structures, such as the tongue and nerves, can complicate surgical resection [61][62]. Additionally, complications from radiation therapy, such as osteoradionecrosis, can significantly impact patient outcomes [22]. The financial burden associated with treatment, including direct costs and long-term rehabilitation, remains a significant concern for patients and healthcare systems alike [19]. In summary, this bibliometric review sheds light on the evolving landscape of mandible bone cancer research. The findings underscore the need for continued investigation into diagnostic and therapeutic strategies to improve outcomes for patients affected by mandibular cancers. Collaborative efforts among researchers, clinicians, and institutions will be essential in addressing the challenges posed by this complex disease.

### Future prospects

The future of mandible bone cancer research and treatment is poised for transformative advancements, driven by innovative therapeutic approaches and technological integration. One promising avenue is the application of immunotherapy, which harnesses the body’s immune system to target cancer cells more effectively. Recent studies have shown that immune checkpoint inhibitors can enhance anti-tumor responses in head and neck cancers, including those affecting the mandible, potentially leading to improved survival rates and reduced recurrence [63]. In addition, gene therapy is emerging as a novel strategy to correct genetic mutations associated with certain types of mandibular cancers. Techniques such as CRISPR-Cas9 have demonstrated the potential to edit genes responsible for tumor growth, offering a personalized approach to treatment [64][65]. Furthermore, advancements in biomaterials and nanotechnology are revolutionizing reconstructive surgery following tumor resection. The development of smart biomaterials that can release therapeutic agents locally, combined with nanoplatforms for targeted drug delivery, may significantly enhance healing and reduce complications like osteoradionecrosis [66][67]. These innovative strategies highlight the need for interdisciplinary collaboration among researchers, clinicians, and engineers to develop comprehensive treatment protocols that improve patient outcomes and quality of life. As research continues to evolve, integrating these cutting-edge technologies into clinical practice will be essential for tackling the challenges posed by mandible bone cancer.

## Conclusion

This bibliometric review provides a comprehensive overview of the research landscape surrounding mandible bone cancer from 1933 to 2024, revealing significant trends in publication and citation metrics. The steady increase in research output reflects growing interest and engagement within the scientific community. While advancements in treatment modalities have improved patient outcomes, challenges persist, particularly regarding the complexity of surgical interventions and the psychological burden of the disease. The identification of key institutions and authors underscores the collaborative nature of this field, which is essential for fostering innovation and improving care. Future research should focus on personalized treatment approaches, enhanced surgical techniques, and the psychosocial dimensions of patient care. By addressing these areas, the field can continue to progress toward more effective management of mandible bone cancer, ultimately benefiting affected individuals and their quality of life.

## Conflict of Interest

The authors declare no potential conflicts of interest with respect to the research, authorship, or publication of this article.
